# The Human Glioma-Associated Oncogene Homolog 1 (GLI1) Family of Transcription Factors in Gene Regulation and Diseases

**DOI:** 10.2174/138920210791233108

**Published:** 2010-06

**Authors:** Hu Zhu, Hui-Wen Lo

**Affiliations:** 1 Department of Surgery, Division of Surgical Sciences, Duke University School of Medicine; 2 Duke Comprehensive Cancer Center, Durham, NC 27705, USA

**Keywords:** GLI1, GLI1∆N, tGLI1, Sonic Hedgehog pathway, Smoothened, CD24, polymorphism, splice variants, cancer.

## Abstract

Sonic hedgehog (Shh) signaling is critically important for embryogenesis and other cellular processes in which GLI transcription factors mediate the terminal effects of the pathway. GLI1, in particular, plays a significant role in human cancers. Consequently, GLI1 and its upstream positive regulator Smoothened (SMO) are important targets of anti-cancer therapy and several SMO-targeted small molecule inhibitors are being evaluated clinically. Emerging exciting evidence reveals a high level of complexity that lies within the GLI1-mediated pathway. For example, a recent study provided evidence linking the polymorphic GLI1 variants Q1100/E1100 to chronic inflammatory bowel diseases. Two recent reports uncovered the existence of two novel human GLI1 isoforms that differ structurally and functionally from the wild-type GLI1 identified over two decades ago. Interestingly, although both are products of alternative splicing, GLI1∆N and tGLI1 (truncated GLI1) isoforms are predominantly expressed in normal and malignant tissues, respectively. In addition to these important discoveries, gene expression profiling studies have identified a number of novel wild-type GLI1 and tGLI1 target genes, linking wild-type GLI1 to tumor progression and therapeutic resistance, and tGLI1 to tumor invasion and migration. In light of these new insights, this review will provide a comprehensive overview on GLI1 polymorphisms and the three members of the GLI1 family of proteins, and their impacts on human diseases, including, cancers.

## 1. INTRODUCTION

Glioma-associated oncogene homolog 1, GLI1, was initially identified as an amplified gene in a human malignant glioma [1] and later, characterized to be a member of the Kruppel family of zinc finger-containing transcription factors [2, 3]. GLI1 and the other two members of the GLI family, namely, GLI2 and GLI3, are the nuclear mediators of the Shh signaling pathway that regulates various aspects of early development of central nervous system [4]. For example, the Shh-GLI1 pathway is required for the differentiation of the floor cell and ventral neurons in the neural tube, granule cell precursor proliferation in the cerebellum, and dorsal brain growth [4-7]. In addition to normal development, the Shh-GLI1 pathway is critically involved in tumorigenesis, cancer growth and cancer stem cell self-renewal [4, 8-10]. 

Shh-mediated signal transduction is activated following binding of the secreted Shh ligand to its receptor Patched (PTCH), a 12-transmembrane protein that inhibits the activity of the seven-transmembrane domain protein Smoothened (SMO). Shh-bound PTCH activates SMO and activated SMO releases GLI1 from cytoplasmic sequestration and, in turn, GLI1 translocates into the cell nucleus to regulate gene expression [9]. GLI1 binds to the consensus GLI1-binding element within its target genes [3], leading to activation of a number of genes that regulate important cellular processes, such as, G1 cell cycle progression, cell proliferation and differentiation, anti-apoptosis, tumor progression, metastasis and tumorigenesis. 

Although mutations are frequently found in other components of the Hedgehog pathway including PTCH and SMO, no mutations have been reported in the GLI1 gene. Human GLI1 gene amplification is also uncommon in most cancers even though it was initially identified as an amplified gene in a cancer cell line. However, the incidence rates for GLI1 amplification vary significantly with the highest frequency (28%) reported in alveolar rhabdomyosarcomas [11]. A number of polymorphic variants of the human GLI1 gene have been reported, with the majority of them localized within the non-coding regions. For those occurring within the coding region of the GLI1 gene, one particular polymorphism at codon 1100 (Q/E) in exon 12 has attracted much attention because of its predictive value for chronic inflammatory bowel diseases [12]. 

Strikingly, more than two decades after the cloning of the coding region of the human GLI1 gene in 1988 [2], two alternatively spliced GLI1 variants were identified by Dr. Peter Zaphiropoulos and colleagues [13] and our laboratory [14]. Structural and functional comparisons of the three GLI1 isoforms have yielded interesting results and will be summarized in details in later sections. In addition to amplification, polymorphism and post-transcriptional splicing, two GLI1 gene transcripts differing in their 5’-untranslated regions due to alternative splicing of untranslated exon 1 were reported [15]. Interestingly, Dahlen and colleagues [16, 17] reported provocative genomic rearrangements, t(7;12)(p21-22;q13-15), that result in a fusion gene product consisting 5’-region of β-actin gene and 3’-region of GLI1 gene. The zinc finger DNA-binding domains of GLI1 were retained in the fusion transcripts. Albeit the β-actin-GLI1 gene fusion was found in soft tissue tumors, it remained unclear with regards to its incidences in other tissues and its physiological effects. Given the fact that significantly more information is available on amplification, polymorphism and post-transcriptional splicing of the human GLI1 gene, the review will focus on these aspects.

## 2. GLI1 GENE AMPLIFICATION

GLI1 was identified as an amplified gene in a human glioblastoma (GBM) cell line, D-259 MG in 1987 [1]. It was first mapped to chromosome 12q13-14.3 by Kinzler *et al. *[1] and later, to the chromosome sub-bands 12q13.3-14.1 [18]. In 1998, coding region of the human GLI1 gene was determined and subsequently characterized to be a member of the Kruppel family of zinc finger protein [2]. Albeit the human GLI1 was initially isolated from a GBM cell line with GLI1 gene amplification, the incidences of GLI1 amplification in human malignant gliomas were found to be relatively low as reported by studies conducted in the late 1980’s and early 1990’s. It is worth noting that these earlier studies utilized a combination of fluorescence *in situ *hybridization and southern blots, which is different than the more advanced genome-wide copy-number analysis. For example, Bigner *et al. *[19] reported one out of 33 (3.3%) primary malignant gliomas to contain amplified GLI1 gene. Wong *et al. *[20] identified only one out of 63 (1.6%) malignant gliomas to carry GLI1 amplification. Forus *et al. *[21] found 2% (2/98) of the primary GBM cohort to contain GLI1 gene amplification. In contrast to these observations, a more recent 2009 study [22] used genome-wide copy number analysis and subsequently identified 22.6% of 31 GBM samples to have amplified GLI1 gene locus. 

GLI1 gene amplification was extensively studied in other types of human cancers. Several studies showed that GLI1 amplification is absent in pediatric medulloblastoma, a common childhood brain cancer [23, 24]. GLI1 amplification was reported in childhood sarcomas but the frequency varies considerably among different types of sarcomas. For example, Roberts *et al*. [25] found the GLI1 gene to be amplified in one out of 13 rhabdomyosarcomas, most common soft-tissue sarcoma found in children, and one out of nine osteosarcomas analyzed. None of the seven Ewing’s sarcomas contained GLI1 amplification [25]. Gordon *et al. *[11] reported 28% of 44 primary tumors and six cell lines of alveolar rhabdomyosarcomas to contain amplified GLI1 gene. Analysis of 37 primary adult sarcomas by Stein *et al. *revealed the absence of GLI1 amplification in this tumor cohort [26]. Lack of GLI1 gene amplification was further reported in uterine leiomyomas [27] and gastrointestinal stromal tumors [28]. In bladder cancer, Simon *et al. *[29] found only 0.4% of 2,317 bladder cancer specimens to express amplified GLI1 gene. 

## 3. GENETIC POLYMORPHISM OF HUMAN GLI1 GENE

Multiple single-nucleotide polymorphisms (SNPs) have been identified in human GLI1 gene. These SNPs are located in the coding region, as well as, introns and 5’- and 3’-untranslated regions of the GLI1 gene. A full spectrum of these SNPs can be found at the NIH-NCBI SNPs database (http://www.ncbi.nlm.nih.gov/projects/SNP/). Albeit many SNPs are present in the human GLI1 gene, the biological understanding of the majority of these SNPs is very limited. The most extensively studied GLI1 SNPs are 2876A→G (codon 933D→G; SNP number rs2228224) and 3376C→G (codon 1100Q→ E; SNP number rs2228226). Interestingly, the first human GLI1 gene sequence isolated from D-259 MG GBM cell line [1] contained codons G933 and E1100 (Genbank accession number NM_005269). These alleles were also identified later by our laboratory [14] using another GBM cell line U87MG (Genbank accession number GQ890670) corroborating the observations made by Kinzler *et al. *[1]. The Q1100 allele was later isolated from a healthy individual [30]. The GLI1 D933 allele was reported in healthy individuals by Hynes *et al. *and Kogerman *et al. *[31, 32]. 

More recent studies conducted in the 2000’s further analyzed larger cohorts of healthy controls for the polymorphic changes of GLI1 gene and provided important information. Wang *et al. *[33] investigated a cohort of 48 healthy Australians and found the G933 allele to be the less frequent allele (45%) compared to the D933 allele (55%). Interestingly, individuals with homozygous G933 only represented 20% of the population analyzed while the majority of the cohort was either heterozygous (50%) or homozygous for D933 (30%). A most recent 2009 study by Lees *et al. *[12] further examined a total of 1374 health Scottish individuals and found the frequencies of G933 and D933 alleles to be 38.3% and 61.7%, respectively. Similar to the findings by Wang *et al. *[33], Lees and the colleagues found that homozygous G933 was the least common (16.3%) compared to heterozygous (44%) or homozygous for D933 (39.7%). Furthermore, Lees *et al. *examined whether associations existed between GLI1 codon 933 polymorphism and the risks of developing ulcerative colitis (UC) and Crohn’s disease (CD). Both diseases belong to chronic inflammatory bowel diseases that are of high prevalence and associated with considerable morbidity. A total of 474 and 335 patients with UC ad CD, respectively, were included in this study. The results, however, indicated that GLI1 codon 933 polymorphism was not significantly associated with increased risks for UC or CD. 

Interestingly, the E1100 variant of the GLI1 protein was found to be less active in its transactivational ability compared to the Q1100 variant [12]. This observation is in line with the fact that Q1100/E1100 is well conserved among different species of mammalian GLI1 proteins and is near the C-terminal transactivation domain (aa 1020-1091) [34]. Moreover, both E and Q variants appear to demonstrate a similar degree of nuclear translocalization as expected, since the nuclear localization signal (NLS) is close to the N-terminus of the GLI1 protein. To determine the frequencies of polymorphic Q1100 and E1100 variants in healthy controls and patients with UC and CD, as well as, the association between GLI1 codon 1100 polymorphism and UC/CD, Lees *et al. *[12] analyzed three large cohorts of Scottish (N=2191), English (N=2337) and Swedish (N=774) populations. In the Scottish cohort, a strong correlation was found between GLI1 E1100 variant and increased risks for both UC and CD. In the English cohort, a positive association was observed between GLI1 E1100 variant and increased risks for developing UC but not CD. No correlations were identified in the Swedish population. In line with these interesting observations, healthy Scottish controls with homozygous low-activity E1100 GLI1 variant only represent 8.9% of the cohort whereas those with the heterozygous genotype and homozygous Q1100 account for 42.9% and 48.3%, respectively, of the study cohort. Corroborating these observations, Wang *et al. *[33] reported a significantly lower frequency (2%) for the homozygous E1100 genotype compared to heterozygous genotype (30%) and homozygous Q1100 (68%) using a cohort of 48 healthy Australian individuals. 

Taken together, GLI1 codon 1100 polymorphism appears to be a predictive SNP for chronic inflammatory bowel diseases in some populations, such as, those of Scotland and England, but not Sweden. These emerging important observations should be extended to *in vitro* and *in vivo* mechanistic studies that investigate the role that the GLI1 E1100 variant plays in the development of chronic inflammatory bowel diseases. In light of the fact that the association between GLI1 E1100 variant and inflammatory bowel diseases differs in three different ethnic groups and varies among two different subclasses of inflammatory bowel diseases, future studies are also needed to understand if other genetic factors cooperate with GLI1 codon 1100 polymorphism in the risk estimate of these diseases. It will be also of importance to further explore the frequency of GLI1 codon 1100 polymorphism in other human diseases, such as cancers, given the interesting fact that the GLI1 E1100 variant is carried by the two GBM cell lines (D-259 MG and U87MG) that have been genotyped thus far [1, 14]. 

## 4. POST-TRANSCRIPTIONAL SPLICE VARIANTS OF HUMAN GLI1 GENE

Somatic mutations are absent in the human GLI1 gene, unlike other components of the Hedgehog pathway, such as, PTCH1 [35-39] and SMO [40-43]. Other members of the GLI family of transcription factors, namely, GLI2 [44] and GLI3 [45-47] have also been shown to undergo somatic mutations and the mutants are associated with cancer risks and other disorders. Albeit the human GLI2 gene results in several alternatively spliced variants with differential transcriptional activity [48, 49], only one GLI1 gene transcript has been reported since two decades ago until very recently. In 2008 and 2009, two alternatively spliced human GLI1 variants were identified, namely, GLI1∆N (Genbank accession number AB239136) by Shimokawa *et al. *[13] and tGLI1 (truncated GLI1; Genbank accession number QB890670) by our laboratory [14]. These two splice variants differ from the wild-type full-length GLI1 both structurally and functionally. It is worthwhile to mention that Kinzler and colleagues [2] has noted in their 1988 breakthrough study that a smaller GLI1 transcript existed in one of the cell lines analyzed. It is likely that the shorter GLI1 transcript observed by these investigators corresponded to GLI1∆N or tGLI1, given their further genomic analysis of the GLI1 gene in this cell line indicated no structural rearrangements.

As shown in Fig. (**1**), the human GLI1 gene is consisted of 12 exons including 5’-untranslated exon 1. Translation starts at ATG at nt +79 within exon 2 and is marked by arrows. The entire wild-type GLI1 coding region spans nt +79 to +3399 that encodes 1107 amino acids. A number of functional domains are located within the wild-type GLI1 gene product. Two degron degradation signals are located at the N- and C-termini of the GLI1 protein, namely, Dn and Dc at aa 77-116 and 464-469, respectively [50]. Two SUFU-binding domains (SU) have been mapped to aa 111-125 and the C-terminus of the GLI1 protein [51]. SUFU-bound GLI1 is retained in the cytoplasm [32]. There are five zinc-finger DNA-binding domains (ZF; aa 235-387) that enable GLI1 to bind to DNA sequence to activate gene expression [2]. Two nuclear localization signals (NLSs) consisted of basically charged residues are present with aa 380-420 and allow for nuclear import of the GLI1 protein. Located at the C-terminus (aa 1020-1091), the transactivation domain is required for the transactivation function of GLI1 [34].

### 4.1. GLI1∆N Isoform

The human GLI1∆N variant is a product of alternative splicing that lacks entire exons 2 and 3, in which the translation starts at aa position 129 [13]. As summarized in Fig. (**1**), the encoded GLI1∆N protein contains a deletion of N-terminal 128 amino acids spanning aa 1-128. Notably, GLI1∆N lacks the N-terminal Dn signal and SUFU-binding domains, but retains zinc-finger domains, NLS, C-terminal Dc signal and SUFU-binding domain, and transactivation domain. This alternative splicing event does not occur to the mouse gli1. Analysis of the GLI1∆N and wild-type GLI1 transcripts in adult human normal tissues revealed their expression levels to be comparable. GLI1∆N expression is undetectable in normal brain [13, 14] and glioblastoma cells [14]. In several tumor cell lines, GLI1∆N expression was generally lower than wild-type GLI1. Expression of GLI1∆N and wild-type GLI1 is equally activated by the SMO agonist. Importantly, GLI1∆N demonstrated a weaker capacity than wild-type GLI1 to activate promoters that are under the control of consensus GLI1-binding sites and promoters of known GLI1 target genes, such as, PTCH and IL1R2 [13]. 

Consistent with the weaker transcriptional activity, GLI1∆N appears to undergo a lower degree of nuclear transport than wild-type GLI1. However, given the fact that GLI1∆N lacks the N-terminal SUFU-binding domain, reduced nuclear transport is somewhat unexpected. Consistent with the loss of one of the two SUFU-binding domains, GLI1∆N nuclear translocalization was impacted to a lesser extent by forced expression of SUFU compared to wild-type GLI1. In summary, the discovery of GLI1∆N by Shimokawa and colleagues [13] opened a new avenue of GLI1 research and warrants future investigations into the role of GLI1∆N in normal development and human diseases. In light of the findings that GLI1∆N is expressed to the extent comparable to wild-type GLI1 in adult normal tissues and that GLI1∆N displays a weaker transcriptional activity, it will be of interest to examine its expression in tissues undergoing development and to investigate its physiological effects on embryonic development. 

### 4.2. tGLI1 Isoform

In an attempt to isolate and clone the human GLI1 cDNA, we unexpectedly found that the human GBM cells express a truncated GLI1 (tGLI1) transcript in addition to the well-characterized wild-type counterpart [14]. Nucleotide analysis of tGLI1 indicates that it contains an in-frame deletion of 123 bases, nt 179-301, that spans entire exon 3 and partial exon 4, and encodes 41 amino acids corresponding to aa 34-74 (Fig. (**1**)). The tGLI1 isoform retains various functional domains of the wild-type GLI1, unlike GLI1∆N that loses N-terminal degron degradation signal and N-terminal SUFU-binding domain. To investigate whether the deletion is a product of genetic alternations, genomic DNAs from glioma cell lines, normal human astrocytes and normal adult peripheral blood leukocytes were extracted, PCR-amplified exons 2-4 and nucleotide sequences of the amplified region determined. The results showed no deletion at the genomic level, thus indicating that the tGLI1 variant is a product of post-transcriptional alternative splicing. 

The majority of GBM cell lines, xenografts and primary specimens expressed comparable levels of tGLI1 and GLI1. In addition to the GBMs, tGLI1 was highly expressed in human breast cancer cells [14] and medulloblastoma cells (unpublished data). In a sharp contrast to GLI1, tGLI1 is undetectable in normal brain tissues and other normal tissues. Consistent with the fact that tGLI1 protein retains functional domains of GLI1, tGLI1 and GLI1 demonstrate similar abilities to transactivate consensus GLI1-binding sites and undergo nuclear translocalization. Importantly, gene expression profiling *via *DNA microarray showed that tGLI1 not only activates GLI1 target genes but also modulates additional set of genes. Specifically, tGLI1 activates PTCH expression to a similar extent compared to GLI1. In tGLI1-expressing GBM cells, the expression of 75 genes were expressed at a significantly higher level and 26 genes more suppressed compared to isogenic counterparts with wild-type GLI1. Based on the results of gene expression profiling, tGLI1 may behave as a gain-of-function GLI1 transcription factor. 

Importantly, tGLI1 but not GLI1 promotes migration and invasion of GBM cells and xenografts. One of the tGLI1 target gene, CD24, was over-expressed in tGLI1- but not GLI1-expressing GBM cells and xenografts. Importantly, expression of CD24 is required for tGLI1-mediated increase of GBM migration and invasion as indicated by transcriptional over-expression and down-regulation studies. The observed critical role that CD24 plays in the migratory and invasive phenotype of GBMs is consistent with its known role in metastasis of breast cancer and other cancers of epithelial origin [52, 53]. Results of protein-DNA binding experiments and promoter analysis further defined CD24 as a direct transcriptional target of tGLI1 but not GLI1 [14]. Deletional studies subsequently determined that a tGLI1-binding element is located within a 0.15-kb region of the human CD 24 promoter. 

The gain of function of tGLI1 in regulating CD24 transcriptional activity and promoting GBM cell migration and invasion, its GBM-specific expression pattern, and the fact that it retains all the functional domains of GLI1, collectively, suggest that tGLI1 may be a more important mediator of GBM cellular physiology and behavior than GLI1. These results are significant given the fact that GBM is the most frequent and deadliest brain cancer in adults and is highly infiltrative and resistant to therapy [54-57]. These findings provide a rationale for investigations for the presence of and function of tGLI1 in other tumors known to have active Hedgehog signaling and to be highly metastatic. The discovery of tGLI1 is, thus, highly significant and is likely to open up novel concepts of the role of GLI1 in tumor biology and may provide the basis for novel treatment strategies. Yet answered by this initial discovery, however, are the mechanisms underlying tGLI1-mediated transcriptional regulation and the impact of tGLI1 on other malignant biology of human cancers and on oncogenesis. 

In summary, recent discoveries of novel human GLI1 isoforms have shed new light on the hedgehog signaling pathway. Given the differential functions displayed by the three GLI1 proteins, alternatively splicing may play a significant role in the functional regulation of GLI1. This is particularly important since genetic alterations GLI1 are very rare. It is thus important to extend our research efforts to finding physiological and pathological effects of the two newly identified GLI1 isoforms. Also unanswered by these discoveries, are the mechanisms that regulate the alternative splicing events. In this regard, gaining an understanding of the regulatory factors that switch on tGLI1 expression in cancerous cells may lead to a means of silencing tGLI1 expression and thereby inhibits tumor invasiveness and migration. 

## 5. TRANSCRIPTIONAL TARGETS OF THE GLI1 FAMILY OF TRANSCRIPTION FACTORS

GLI1 protein binds to a 9-base-pair DNA element 5’-GACCACCCA-3’ within its target genes to regulate gene expression [3]. Through gene regulation, the GLI1 family of proteins regulates a number of important cellular processes, such as, neural development, cell proliferation, oncogenesis, survival, epithelial-mesenchymal transition (EMT), migration, invasion and metastasis [58] (Fig. (**2**)). As positive and negative feedbacks, GLI1 protein respectively activates its own expression and that of PTCH1 [59]. Specifically, GLI1 binds to an inverted GLI1-binding site, 5’-TGGGTGGTC-3’ in the PTCH1 promoter leading to gene activation. The tGLI1 protein activates PTCH1 expression to a similar degree as the wild-type GLI1 [14]. 

The first identified GLI1 target gene was hepatocyte nuclear factor-3β, HNF-3β [60]. HNF-3β is  a  winged helix transcription factor that plays a critical role in the development of floor plate which is essential for ventral pattern formation and axonal guidance within the neural tube of vertebrate embryos. GLI1 directly binds to a GLI1-binding site (5’-GAACACCCA-3’) within the HNF-3β promoter to activate HNF-3β gene expression, linking the Hedgehog pathway to neuronal development [60]. In addition to HNF-3β, GLI1 regulates expression of other transcription factors, including, FOXM1 [61] and Jun [62]. FOXM1 belongs to the Forkhead box (FOX) family of proteins that regulates genes involved in cell proliferation, differentiation, aging, and transformation. Teh *et al. *[61] showed that forced expression of GLI1 in primary keratinocytes and other cell lines caused a significant elevation of FOXM1 transcripts and promoter activity, albeit it is unknown whether a GLI1-binding site is located in the FOXM1 promoter. Most recently, Laner-Plamberger *et al. *[62] reported that GLI1 directly binds to a GLI1-binding site (5’-GGCCCCCCA-3’) within the human Jun promoter, leading to its transcriptional activation in the skin. 

GLI1 mediates cell proliferation *via *activating expression of several positive regulators of cell-cycle progression, such as, inhibitors of Rb tumor suppressor, cyclins. For example, GLI1 activates expression of the human cyclin D2 gene following binding to a GLI1-binding site, 5’-CACCACCCA-3’, in the cyclin D2 gene promoter [63]. The Drosophila homolog of GLI1, Ci, directly associates with promoters of cyclin D and cyclin E to upregulate their transcription [64]. In contrast, GLI1 has been shown to also activate insulin-like growth factor binding protein-6 (IGFBP-6) [63] that is anti-proliferative. IGFBP-6 differs from other IGFBPs for its IGF-II binding specificity and subsequent inhibition of IGF-II actions [65]. IGFBP-6 appears to inhibit growth of IGF-II-dependent cancers, including rhabdomyosarcoma, neuroblastoma and colon cancer. 

Accumulating evidence suggests an essential role that GLI1 plays in tumor progression and metastasis. GLI1 induces expression of E-cadherin repressor, Snail, although the exact binding site remains to be identified [66, 67]. Snail, Slug and TWIST belong to a host of EMT regulators that primarily function as transcriptional repressors of cell-cell junction protein, E-cadherin [68]. Loss of cell-cell junction is an essential step of EMT. Furthermore, GLI1 appears to upregulate a chemokine receptor CXCR4 that plays an important role in metastasis [69]. Plus, GLI1 up-regulates expression of rat, mouse and human osteopontin genes [63, 70]. Specifically, GLI1 binds to the GLI1-binding site (5′-TGCTGAATGCCCATCCC-3’) within the human osteopontin gene [70]. Osteopontin is a glycoprotein expressed by various tissues and cells as secreted and intracellular proteins [71]. Secreted osteopontin interacts with integrins and CD44 to mediate cell adhesion, migration and tumor invasion. Overexpression of osteopontin is associated with invasion and metastasis of melanoma and squamous cell carcinoma cells. In line with this notion, Das *et al. *[70] found that GLI1-mediated upregulation of osteopontin leads to increased migration, invasion and proliferation of melanoma cells and increased metastasis of melanoma xenografts in nude mice. Conversely, transcriptional knockdown of GLI1 reduces osteopontin expression, leading to reduced metastasis. Our laboratory showed that tGLI1 protein promotes cell migration and invasion by binding to a 0.15-kb region (nt -294 to -141) of the CD24 promoter and activating CD24 expression. CD24 has been shown to recruit adhesion molecules to lipid rafts, thereby, contributing to tumor cell migration, dissemination and metastasis [52, 53]. Together, the emerging evidence indicates that the GLI1 family of proteins may play an important role in tumor progression and metastasis. 

Additional evidence suggests a potential involvement of GLI1 in therapeutic response of human cancers. For example, GLI1 activates expression of anti-apoptotic protein bcl-2 in keratinocytes [72]. A functional GLI1-binding site, 5’-GACCACCAA-3’, is located in the bcl-2 gene promoter. The functional consequences of GLI1-mediated bcl-2 up-regulation, such as, cell survival, remain to be elucidated. In medulloblastomas, GLI1 has been linked to increased expression of tumor suppressor p53 and O-6-methylguanine-DNA methyltransferase, MGMT [69]. While GLI1 may indirectly activate p53, it may directly activate the MGMT gene given that the MGMT gene promoter contains a putative GLI1-binding site. Although the study by Yoon *et al. *did not provide direct evidence linking GLI1 to therapeutic resistance, both p53 and MGMT have known involvement in drug resistance. In gliomas, the presence of p53 results in higher resistance to cisplatin than when the gene is mutated and/or functionally inactive [73]. Abrogation of the wild-type p53, e.g. *via *antisense oligonucleotides, sensitizes tumor cells to cisplatin. This is consistent with the fact that p53 activates expression of cisplatin-metabolizing enzyme, GSTP1 [74]. Similar observations of wild-type p53 conferring drug resistance or decreased drug sensitivity have also been made in other cancer types, such as, breast, bladder, and ovarian carcinomas [75-77]. MGMT is known to repair O-6-alkylguanine DNA lesions caused by an anti-cancer alkylator, temozolomide [78]. The impact of GLI1-mediated upregulation of p53 and MGMT on therapeutic response in human cancers remains to be investigated. 

## Figures and Tables

**Fig. (1). Structures of the three members of the human GLI1 family of proteins.**
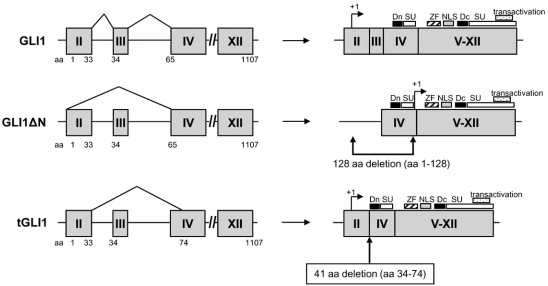
The human GLI1 gene is consisted of 12 exons including 5’-untranslated exon 1. Exons are indicated as gray boxes while introns are shown by lines. Translation starts at ATG at nt +79 within exon 2 and is marked by arrows. The entire wild-type GLI1 coding region spans nt +79 to +3399 that encodes 1107 amino acids. The human GLI1∆N variant is a product of alternative splicing that lacks entire exons 2 and 3, in which the translation starts at position 129. Thus, encoded GLI1∆N protein contains a deletion of N-terminal 128 amino acids spanning aa 1- 128, lacking the N-terminal Dn signal and SUFU-binding domains (SU), but retains zinc-finger domains (ZF), NLS, C-terminal Dc signal, C-terminal SUFU-binding domain and transactivation domain. The tGLI1 transcript is also a product of alternative splicing that lacks entire exon 3 and partial exon 4, resulting in a deletion of 41 codons corresponding to aa 34-74. tGLI1 retains intact degron degradation signals (Dn and Dc; soild bars), SUFU-binding domain (clear bars), Zinc finger domains (back-slash bar), NLS (gray bar), and transactivation domain (dotted bar).

**Fig. (2):**
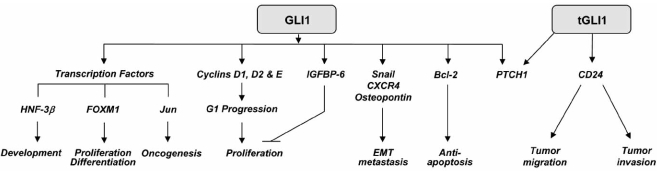
The GLI1 family of transcription factors regulates expression of a host of target genes that thereby mediates a number of important cellular processes.

## ABBREVIATIONS

aa: = Amino acid
CD: = Crohn’s disease
CXCR4: = C-X-C motif receptor 4
EMT: = Epithelial-mesenchymal transition
GBM: = Glioblastoma multiforme
GLI1: = Glioma-associated oncogene homologue 1
GSTP1: = Glutathione S-transferase P1
HNF-3β: = Hepatocyte nuclear factor-3β
IGF-II: = Insulin-like growth factor-II
IGFBP-6: = Insulin-like growth factor binding protein-6
IL1R2: = Interleukin-1 receptor 2
MGMT: = O-6-methylguanine DNA methyltransferase
NLS: = Nuclear localization signal
PTCH: = Patched
Rb: = Retinoblastoma
Shh: = Sonic hedgehog
SMO: = Smoothened
SNP: = Single-nucleotide polymorphism
SUFU: = Suppressor-of-Fused
UC: = Ulcerative colitis
